# The prognostic value of peripheral ischemic microvascular reserve in sepsis is not related to calcitonin gene-related peptide or substance P

**DOI:** 10.5935/0103-507X.20220102-en

**Published:** 2022

**Authors:** Ana Carolina de Miranda, Fernanda do Carmo De Stefani, Hipólito Carraro Júnior, Alain Márcio Luy, Luiz Eduardo Nunes Ferreira, Luis Gustavo Morello, Igor Alexandre Cortês de Menezes

**Affiliations:** 1 Department of Internal Medicine, Hospital de Clínicas, Universidade Federal do Paraná - Curitiba (PR), Brazil.; 2 Intensive Care Unit, Hospital de Clínicas, Universidade Federal do Paraná - Curitiba (PR), Brazil.; 3 Laboratory of Inflammation and Immunology, Universidade de Guarulhos - São Paulo (SP), Brazil.; 4 Fundação Oswaldo Cruz - Curitiba (PR), Brazil.

**Keywords:** Sepsis, Microcirculation, Ischemia, Hyperemia, Perfusion index, Neuropeptides

## Abstract

**Objective:**

To evaluate the mechanisms attributed to the prognostic value of peripheral ischemic microvascular reserve in patients with sepsis.

**Methods:**

This observational cohort study enrolled 46 consecutive septic patients in the intensive care unit between November 2020 and October 2021. After fluid resuscitation, the peripheral ischemic microvascular reserve was evaluated using the association of postocclusion reactive hyperemia with the peripheral perfusion index. Additionally, peripheral venous blood samples were used to evaluate the neuropeptide calcitonin gene-related peptide and substance P levels in the upper limb before and immediately after postocclusion reactive hyperemia

**Results:**

There was no statistically significant correlation (p > 0.05) between basal values (pg/mL) or variations from neuropeptide levels (%) and the peripheral ischemic microvascular reserve (%).

**Conclusion:**

Although calcitonin gene-related peptide and substance P may have a prognostic role in sepsis, these neuropeptides do not appear to contribute to peripheral ischemic microvascular reserve.

## INTRODUCTION

Sepsis is characterized by a widespread and deregulated immune-metabolic host response induced by an infection that results in potentially fatal organ dysfunction.^(1)^ Even though there have been scientific advances in its pathophysiological understanding and management, this syndrome affects millions of patients annually and remains a critical condition associated with high mortality rates and short- and long-term morbidity.^(2)^

Under pathological conditions of sepsis, persistent microcirculatory disorders, such as arteriolar hyporesponsiveness, and capillary dysfunctions have been associated with organ dysfunction and the worst prognosis.^(3)^ Therefore, safe and validated methods to assess vascular reactivity at the bedside are sought in sepsis. Recent findings demonstrated the safety and robust prognostic value of combining the noninvasive methods peripheral perfusion index (PPI) and postocclusive reactive hyperemia test (PORH) to evaluate the percent change of blood flow in response to flow-dependent tissue hypoxia.^(4,5)^ Thus, the combined PORH/PPI could estimate the microvascular reactivity and reserve of the examined tissue in septic shock.

Interestingly, a paradoxical observation was made when using PORH/PPI as a measurement method for patients with septic shock: those with higher peripheral ischemic microvascular reserve (PIMR) had a worse prognosis.^(5)^ However, there is no clear evidence in the literature to elucidate this unexpected finding related to PIMR or its potential prognostic role. A possible hypothesis is based on the role of the sensory neuropeptides calcitonin gene-related peptide (CGRP) and substance P (SP). These neuropeptides constitute part of the mechanisms responsible for cutaneous vasoregulation^(6)^ and immune modulation^(6)^ and have prognostic value in sepsis.^(7-9)^ Furthermore, experimental evidence strongly suggests sensory nerve involvement in the cutaneous postischemic response in healthy conditions.^(10,11)^ Thus, we hypothesized that the elevation of neuropeptides after the POHR/PPI test could contribute to the high microvascular reserve in more severely ill patients and concomitantly indicate a more significant immune dysregulation in these patients. Hence, to answer this question, the present research investigated a subgroup of a validation study of POHR/PPI as a method of microvascular evaluation of septic patients.

## METHODS

### Study design, setting and participants

This observational cohort study was conducted in the 15-bed Brazilian intensive care unit (ICU) between November 2020 and October 2021. All survivor participants or their legal representatives provided written informed consent, except in the case of the patient’s death, in which case the written informed consent was waived. The research was approved by The Human Research Ethics Committee of the *Complexo Hospitalar de Clínicas* of the *Universidade Federal do Paraná* (CHC-UFPR), protocol 4.754.428/2020.

Consecutive adult patients (18 years or older) with a diagnosis of sepsis admitted to the ICU or within 24 hours after its onset in patients previously admitted for other causes were considered eligible for inclusion in the study. The exclusion criteria to minimize potential confounding factors or risks of possible hemorrhagic and ischemic complications of procedures were pregnancy, severe hepatopathy (Child-Pugh class C), severe coagulopathy (platelets < 20,000/mm^3^, the international normalized ratio - RNI > 2.0, or activated partial thromboplastin time - aPTT > 70 s), the presence of severe active bleeding, infective endocarditis, inaccessible perfusion assessment (severe hypothermia; Raynaud’s syndrome, peripheral arterial occlusive disease), and refusal of the patient to participate in the study.

### Clinical definitions

According to the current consensus on sepsis (2016), this syndrome is identified as the presence of an infection associated with an acute change in the Sequential Organ Failure Assessment (SOFA) score of two points or more.^(1)^ Septic shock consists of a subgroup of sepsis cases wherein, despite adequate resuscitation with fluids, patients have elevated serum lactate concentrations ≥ 2mmol/L associated with hypotension requiring vasopressors to achieve the target for mean arterial blood pressure (MAP) above 65mmHg.^(1)^

### Study protocol

All selected patients were conducted following a local institution’s recommendations adapted from the Surviving Sepsis Campaign guidelines.^(12)^ The management started as soon as sepsis was detectable. First, if there was a high likelihood of this syndrome, antimicrobials were administered within the first hour of recognition after collecting blood and suspicious focus culture. Second, in cases of signs of hypoperfusion or septic shock, 30mL/kg of balanced crystalloid fluids was administered at the discretion of the patients’ physicians over the first 3 hours of sepsis diagnosis. Additionally, according to the criteria of the physician, if there was an individual clinical indication, resuscitation was continued until there was a lack of reaction to passive-leg raising (the cutoff value was an increase in cardiac output of 13% to discriminate fluid responders) or no respiratory variance of inferior vena cava diameter (the cutoff of 18%). If MAP persisted less than 65mmHg, norepinephrine was used to obtain MAP ≥ 65mmHg. Vasopressin was the drug of choice for association with noradrenaline in refractory cases. The hemodynamic goals were MAP ≥ 65mmHg, urine output > 0.5mL/kg/h and central venous oxygen saturation (ScvO_2_) > 70%.

The assessment of septic patients occurred within 24 h after proper hemodynamic resuscitation, which was established by stable macrohemodynamics at the end of this period. The data collected during the study included demographic characteristics, medical history, infection source and comorbidities, SOFA scores, and Acute Physiology Chronic Health Evolution II (APACHE II) scores. In addition, all hemodynamic parameters (if available), neuropeptide levels, and peripheral variables were measured between 6 and 24 hours of sepsis diagnosis. In addition, intensivists were blinded to peripheral ischemic microvascular reserve variables to avoid possible treatment interference. Finally, the patients were followed for 28 days of sepsis diagnosis or hospital release.

### Evaluation of peripheral ischemic microvascular reserve

Peripheral ischemic microvascular reserve was assessed using a combination of the PPI and PORH tests. The PPI corresponds to a parameter derived from the photoelectric plethysmography signal of a pulse oximeter, calculated as the pulsatile blood flow ratio (arterial blood) to nonpulsatile blood flow (venous blood, capillary, other tissues, and bones) nail bed from the emission of two sources with two different wavelengths (660 and 940nm).^(13)^ Its value represents an indicator of peripheral vasomotor tone and peripheral perfusion.^(14)^ This variable was measured after proper hemodynamic resuscitation by attaching a pulse oximeter probe (MINDRAY, Shenzen, China). First, after signal stabilization, the PPI values were recorded every 30 seconds for 5 minutes, and the average of the values was calculated to determine the PPI basal value. Subsequently, a test of the microvascular reactivity, called the PORH test,^(15)^ was performed: the cuff of a sphygmomanometer was inflated around the homolateral upper limb at 50mmHg above systolic pressure for 3 minutes. After deflation of the sphygmomanometer cuff, PPI values were recorded every 30 seconds for 5 minutes, and the higher value corresponded to the PPI peak value. Reactive hyperemia was established by changes in blood flow verified using PPI values. Finally, the estimation of the maximum change in blood flow in response to tissue hypoxia (peripheral ischemic microvascular reserve) was established by the ∆ PPI peak (%), which was calculated using the following formula:


$$\Delta \text{ PPI peak }=\frac{\text{ PPI peak }-\text{ PPI basal }}{\text{ PPI basal }}100\%$$

### Evaluation of neuropeptide levels

Venous blood samples were collected within the first 24 hours of sepsis diagnosis after appropriate hemodynamic resuscitation to determine serum concentrations of the neuropeptides CGRP and SP. Approximately 5mL of venous blood was collected from the upper limb evaluated in the forearm region before and immediately after the PORH test. These samples were deposited into test tubes containing ethylenediaminetetraacetic acid (EDTA) and subsequently centrifuged at 1200 relative centrifugal force (RCF) at 4°C for 10 minutes. Approximately 1mL of plasma was frozen at -80°C. The samples were transported between CHC-UFPR - Curitiba, PR, to the Laboratory of Inflammation and Immunology at *Universidade Guarulhos*, SP, in refrigerated boxes containing dry ice.

The presence of the neuropeptides SP and CGRP in human blood samples was measured by ELISA. Substance P and CGRP were quantified using the Substance P Parameter Assay Kit (R&D Systems, Minneapolis, MN, USA) and Human Calcitonin Gene-Related Peptide ELISA Kit (MyBioSource, San Diego, CA, USA) according to the manufacturer’s instructions. A competitive binding ELISA was used to determine the SP concentration. Briefly, 50µL of the samples were added to 96-well plates and incubated with monoclonal antibodies for SP and conjugated to horseradish peroxidase. The plates were incubated for 3 hours, allowing the binding between the monoclonal antibody and the goat anti-mouse antibody coated onto the microplate. Subsequently, the plates were washed, and the substrate solution was added to the wells to determine the bound enzyme activity. The absorbance measurement was established in a microplate reader set at 405nm wavelength with wavelength correction set at 570nm. The SP concentration was expressed in pg/mL.

Calcitonin gene-related peptide analyses were performed using the double antibody sandwich technique. Briefly, 100µL of the samples were added to 96-well plates and incubated with CGRP monoclonal antibody for 90 min. Next, the plates were washed, and 100µL of biotinylated CGRP antibody was added to each well. Finally, the plates were incubated for 60 minutes. Subsequently, avidin-peroxidase was added, and the absorbance was measured in a microplate reader set at 450nm (630nm wavelength correction). The CGRP concentration in the samples was expressed in pg/mL, established by a correlation between the absorbance data and the calibration curve.

The variation in neuropeptide levels in response to tissue hypoxia was established using the following formulas:


$$\Delta \text{ CGRP }=\frac{\text{ CGRP pos ischemic }-\text{ CGRP pre ischemic }}{\text{ CGRP pre ischemic }}100\%$$

$$\Delta \text{ SP }=\frac{\text{ SP pos ischemic }-\text{ SP pre ischemic }}{\text{ SP pre ischemic }}100\%$$

The ambient bedside temperature was controlled at 22ºC. The medical procedure was made in a supine decubitus and performed in the upper limb without an intra-arterial catheter for MAP measurement.

### Outcomes

The primary outcome was the correlation between the peripheral ischemic microvascular reserve (∆ PPI peak, %) and variation in the neuropeptide CGRP and SP levels (∆ neuropeptide levels, %) from the first day of sepsis diagnosis after fluid resuscitation. The secondary outcomes included the correlation between basal venous neuropeptide CGRP and SP levels and the PPI basal values, the correlation between basal venous neuropeptide CGRP and SP and the peripheral ischemic microvascular reserve (∆ PPI peak, %), and the correlation between basal venous neuropeptide CGRP and SP and lactate levels within 24 h of sepsis diagnosis after fluid resuscitation.

### Analytical approach

The Shapiro-Wilk test was utilized to determine the normality of the sample. Parametric data are represented as the means ± standard deviation, while the medians and interquartile range (IQR) were used for nonparametric data. Percentages represent proportions. Correlation tests between the continuous variables of ∆ PPI peak (%) and ∆ neuropeptide levels (%), between venous neuropeptides CGRP and SP lactate levels pre-PORH and the PPI basal values, and between venous neuropeptides CGRP and SP lactate levels pre-PORH and the peripheral ischemic microvascular reserve (∆ PPI peak, %) were performed using the Spearman test. The Wilcoxon signed-rank test compared neuropeptide levels between the first and second measurements. The significance of the results was determined by a p value < 0.05. All reported p values are two-sided. IBM Statistical Package for the Social Science (SPSS) 23 and GraphPad Prism 6 programs were used for all analyses.

The calculation of sample size was established based on the pilot study. We estimated a sample size of 38 patients to find at least a moderate correlation (r = 0.5), with a sample power of 90%. Because the neuropeptide levels are nonparametric variables, an addition of 15% was made,^(16)^ giving an overall sample of 46 patients. The alpha error chosen was 0.05, and the p values were corrected for multiple comparisons.

This research followed the STROBE guidelines for reporting results.

## RESULTS

**Figure 1:**
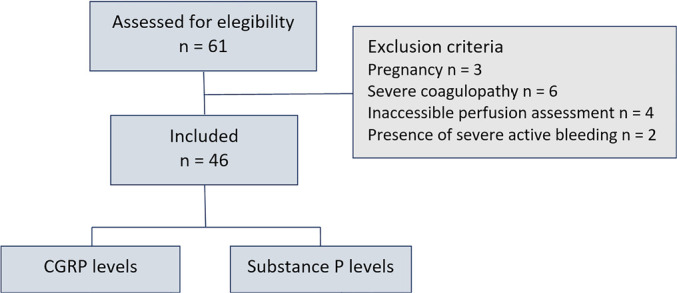
Flowchart of the study.

Forty-six patients were included during the study period after proper fluid resuscitation and had subsequent peripheral ischemic microvascular reserve evaluation associated with the analyses of pre- and postischemic SP and CGRP levels (Figure 1). The clinical-demographic and hemodynamic data of all patients are listed in table 1. In general, these data describe a heterogeneous critically ill population, a typical finding of sepsis. Among the patients studied, the average age was 57 years, most were female (70%), and their main comorbidities were hypertension (52%) and diabetes (30%). Pulmonary (52%) and abdominal (n = 23) were the primary septic sources. Most patients identified the etiologic factor using cultures (85% of the cases), moderate severity scores (SOFA and APACHE II), altered C-reactive protein levels, and procalcitonin. At the PIMR assessment, the global hemodynamic variables were within acceptable ranges.

Sixty-one patients were considered eligible for the study. Fifteen patients were excluded. Forty-six patients were included after proper fluid resuscitation and had subsequent peripheral ischemic microvascular reserve evaluation associated with the analyses of pre- and postischemic substance P and calcitonin gene-related peptide levels. CGRP - calcitonin gene-related peptide.

**Table 1:** The demographic, clinical, and hemodynamic characteristics of septic patients after fluid resuscitation

Parameters	
Clinical	
Age (years)	57 (13)
Sex	
Men	14 (30)
Women	32 (70)
Comorbidities	
*Diabetes mellitus*	14 (30)
Hypertension	24 (52)
Chronic kidney disease	5 (11)
Heart failure	2 (4)
Liver failure	3 (7)
Cerebral vascular disease	3 (7)
Chronic pulmonary disease	10 (22)
Cancer	2 (4)
Immunosuppression	8 (17)
Source of infection	
Respiratory	31 (67)
Abdominal	7 (15)
Urinary	1 (2)
Others	7 (15)
Any microorganism in cultures	39 (85)
Confirmed bloodstream infection	18 (39)
Scores and biomarkers at ICU admission	
SOFA^*^	9 (3)
APACHE II†	25 (9)
CRP (mg/dL)	20 (13)
Procalcitonin (ng/mL)	44/1 (0.2 - 5.8)
Hemodynamic data after resuscitation	
MAP (mmHg)	86 (14)
Heart rate	91 (22)
ScvO_2_ (%)	21/76 (8)
Pv-aCO_2_ (mmHg)	21/7 (6)
Arterial lactate (mmol/L)	43/1.9 (1.4 - 2.4)
Urine output) (mL/kg/h)	47/0.6 (0.4)
Vasoactive drugs use	26 (57)
Norepinephrine dose (µg/kg/min)	0.2 (0.3)
Vasopressin use	6 (13)
Peripheral perfusion	
Prolonged CRF (> 3s)	10 (22)
Altered PPI (< 1.4)	10 (22)
Neuropeptides basal dosage	
CGRP (pg/mL)	27 (31)
Substance P (pg/mL)	153 (21 - 376)

ICU - intensive care unit; SOFA - Sequential Organ Failure Assessment; APACHE - Acute Physiology and Chronic Health Evaluation; CRP - C-reactive protein; MAP - mean arterial pressure; ScvO_2_ - central venous oxygen saturation; Pv-aCO_2_ - venous to arterial carbon dioxide difference; CRF - capillary refill time; PPI - peripheral perfusion index; CGRP - calcitonin gene-related peptide;

* Range, 0 to 24: higher scores are associated with the intensity of organ dysfunction and a higher risk of in-hospital death.^(17)^ † Range, 0 to 71: higher scores are associated with the intensity of illness and a higher risk of in-hospital admission.^(17)^ Results expressed as mean ± standard deviation; n (%); n/median (interquartile range) and n/mean ± standard deviation.

**Figure 2:**
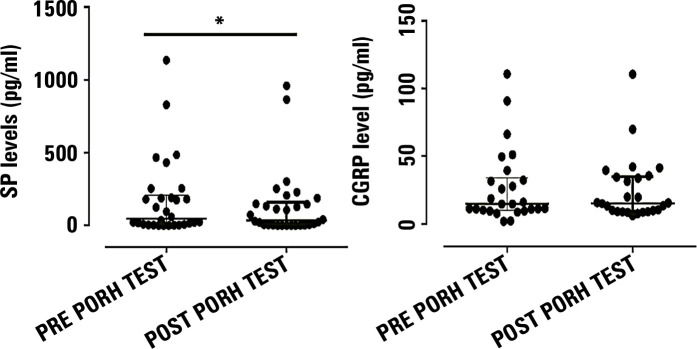
Comparison between neuropeptide levels pre- and post-PORH test. (A) Substance P levels were significantly reduced after the PORH test; (B) Calcitonin gene-related peptide levels did not significantly change after the PORH test (p > 0.05). * p < 0.01

**Figure 3:**
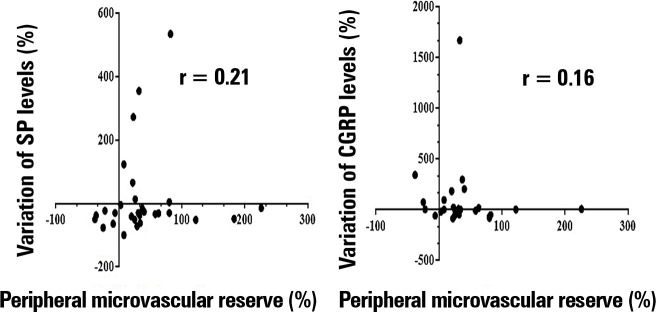
Correlation between the variation in neuropeptide levels pre- and post-postocclusive reactive hyperemia test (∆ neuropeptide levels, %) and the peripheral ischemic microvascular reserve (∆ PPI peak, %). There was no significant correlation between the peripheral ischemic microvascular reserve and variation in the neuropeptide calcitonin gene-related peptide and substance P levels within the first 24 hours of sepsis diagnosis (p > 0.05).

**Figure 4:**
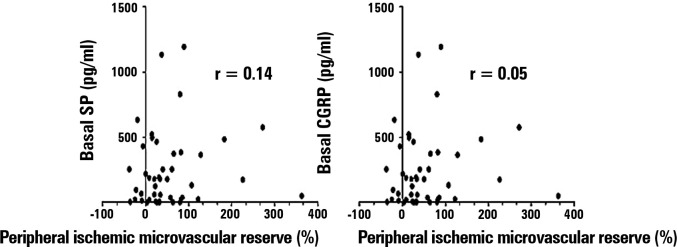
Correlation between the basal levels of the neuropeptides (pg/mL) and the peripheral ischemic microvascular reserve (∆ PPI peak, %). There was no significant correlation between the peripheral ischemic microvascular reserve and basal levels of the neuropeptides calcitonin gene-related peptide and substance P within the first 24 hours of sepsis diagnosis (p > 0.05).

As shown in figure 2, we analyzed the levels of neuropeptides before and after the PORH test. There were statistically significant differences in SP levels (p < 0.01), with the median pretest value higher (46.7; IQR 13.7 - 180.6) than the posttest value (35; IQR 4.9 - 133.5). However, there were no significant differences (p = 0.86) between pre- (14.6; IQR 10.8 - 31.5) and post-CGRP values (15.2; IQR 9.7 - 33.8). Additionally, as demonstrated in figure 3, this study found no significant correlation between the peripheral ischemic microvascular reserve (∆ PPI peak, %) and variation in the neuropeptide CGRP (p = 0.41) and SP (p = 0.26) levels (∆ neuropeptide levels, %) within the first 24 hours of sepsis diagnosis after appropriate hemodynamic resuscitation. Moreover, there was no significant correlation between the peripheral ischemic microvascular reserve (∆ PPI peak, %) and basal levels of the neuropeptides CGRP (p = 0.71) and SP (p = 0.33) (Figure 4).

SP - substance P; CGRP - calcitonin gene-related peptide; PORH - postocclusive reactive hyperemia test.

SP - substance P; CGRP - calcitonin gene-related peptide; PPI - peripheral perfusion index.

SP - substance P; CGRP - calcitonin gene-related peptide; PPI - peripheral perfusion index.

The present study also sought to evaluate a possible association between basal neuropeptide levels and peripheral perfusion but observed no significant correlation between the basal PPI and basal levels of the neuropeptides CGRP (p = 0.95) and SP (p = 0.82). Finally, we found no correlation between lactate level, an accepted marker of tissue hypoperfusion, and the basal neuropeptides CGRP (p= 0.66) and SP (p = 0.92).

## DISCUSSION

Current evidence has demonstrated the ability of peripheral ischemic microvascular reserve measurements to predict the 28-day mortality rate in septic shock patients.^(5)^ Contrary to the behavior of other microvascular territories, a better fingertip ischemic microvascular reserve was associated with higher mortality, thus suggesting a functional rather than a structural mechanism.^(5)^ Nevertheless, no clear evidence has elucidated the paradoxical finding involved in the high PIMR or its potential prognostic role.

The skin corresponds to one of the most accessible organs in the human body, allowing clinicians to evaluate cutaneous microvascular reactivity with noninvasive bedside parameters, such as the PPI associated with the PORH test. Although some mechanisms, such as the roles of nitric oxide (NO) and prostacyclin (PGI-2), seem to be crucial to vasomotor dysfunction in sepsis,^(18)^ the inhibition of these substances does not appear to interfere with reactive hyperemia in the skin.^(19,20)^ Concerning the physiological mechanisms involved in skin microvascular reactivity, evidence has shown that the expression and release of neuropeptides via sensory nerve fibers and substances derived from cytochrome epoxygenases appear to play an essential role in the peak and timing of hyperemia in healthy individuals.^(21,22)^ Moreover, in addition to its performance in cutaneous vasoregulation,^(6)^ robust evidence has demonstrated its value in predicting sepsis mortality.^(7-9)^

The motivation of this study was to investigate the role of the neuropeptides SP and CGRP in the ischemic skin response of septic patients. These neuropeptides have an immune-modulating function^(6)^ and can simultaneously be released after the occlusion test,^(11)^ theoretically explaining the functional mechanism linking a high reserve and higher mortality. In this way, a microhemodynamic test could theoretically be used to indicate a phenotype of more severe septic patients, likely due to more significant immune dysregulation. Unfortunately, our findings did not corroborate this hypothesis. Contrary to expectations, posttest plasma CGRP levels remained similar to pretest levels. Additionally, SP levels were reduced after the test. However, it is well known that several enzymes, including neutral endopeptidase, dipeptidyl aminopeptide IV, and angiotensin-converting enzyme, degrade SP.^(23)^ Therefore, some of these peptidases may have increased activity during the hypoxic-ischemic stimulus of the test in septic patients. In this sense, other studies are needed to confirm this statement.

However, some limitations should be considered before completely ruling out this possibility. First, the time of assessment and measurement was not ideal, since these mediators could have been released later. Second, it was impossible to obtain different dosages of neuropeptides over time to assess a response curve compared to the reserve curve. This limitation is corroborated by one of the interventional studies, which, through the infusion of these neuropeptides into the skin by microdialysis in healthy subjects, observed potent and lasting vasodilation in the case of CGRP and a milder and short duration associated with plasma leakage for SP.^(24)^ Thus, the comparison of maximum response peaks may not fit the hypothesis. Third, the measurement of neuropeptides via venous blood may not represent the measurement evaluated in capillaries. However, our study used an intraindividual comparative analysis, which reduced the possible influence of the mentioned collection sites. Finally, in this study, the evaluation of ischemic peripheral microvascular reserve associated with the dosage of neuropeptides in septic patients was not compared with healthy patients, thus limiting the conclusions.

## CONCLUSION

In conclusion, although calcitonin gene-related peptide and substance P may have a prognostic role in sepsis, these neuropeptides do not appear to contribute to peripheral ischemic microvascular reserve.

## References

[1] Singer M, Deutschman CS, Seymour CW, Shankar-Hari M, Annane D, Bauer M (2016). The Third International Consensus Definitions for Sepsis and
Septic Shock (Sepsis-3). JAMA.

[2] Rudd KE, Johnson SC, Agesa KM, Shackelford KA, Tsoi D, Kievlan DR (2020). Global, regional, and national sepsis incidence and mortality,
1990-2017: analysis for the Global Burden of Disease Study. Lancet.

[3] Charlton M, Sims M, Coats T, Thompson JP (2017). The microcirculation and its measurement in
sepsis. J Intensive Care Soc.

[4] Menezes IA, Cunha CL, Carraro Júnior H, Luy AM (2018). Perfusion index for assessing microvascular reactivity in septic
shock after fluid resuscitation. Rev Bras Ter Intensiva.

[5] Menezes IA, Cunha CL, Júnior HC, Luy AM (2019). Increase of perfusion index during vascular occlusion test is
paradoxically associated with higher mortality in septic shock after fluid
resuscitation: a prospective study. Shock.

[6] Schlereth T, Schukraft J, Krämer-Best HH, Geber C, Ackermann T, Birklein F (2016). Interaction of calcitonin gene related peptide (CGRP) and
substance P (SP) in human skin. Neuropeptides.

[7] Beer S, Weighardt H, Emmanuilidis K, Harzenetter MD, Matevossian E, Heidecke CD (2002). Systemic neuropeptide levels as predictive indicators for lethal
outcome in patients with postoperative sepsis. Crit Care Med.

[8] Lorente L, Martín MM, Pérez-Cejas A, Ferreres J, Solé-Violán J, Labarta L (2017). Sustained low serum substance P levels in non-surviving septic
patients. Int J Mol Sci.

[9] Lorente L, Martín MM, Almeida T, Hernández M, Ferreres J, Solé-Violán J (2015). Association between serum substance P levels and mortality in
patients with severe sepsis. J Crit Care.

[10] Larkin SW, Williams TJ (1993). Evidence for sensory nerve involvement in cutaneous reactive
hyperemia in humans. Circ Res.

[11] Lorenzo S, Minson CT (2007). Human cutaneous reactive hyperaemia: role of BKCa channels and
sensory nerves. J Physiol.

[12] Evans L, Rhodes A, Alhazzani W, Antonelli M, Coopersmith CM, French C (2021). Surviving sepsis campaign: international guidelines for
management of sepsis and septic shock 2021. Intensive Care Med.

[13] Lima A, Bakker J (2005). Noninvasive monitoring of peripheral perfusion. Intensive Care Med.

[14] Lima AP, Beelen P, Bakker J (2002). Use of a peripheral perfusion index derived from the pulse
oximetry signal as a noninvasive indicator of perfusion. Crit Care Med.

[15] Roustit M, Cracowski JL (2013). Assessment of endothelial and neurovascular function in human
skin microcirculation. Trends Pharmacol Sci.

[16] Fujii T, Luethi N, Young PJ, Frei DR, Eastwood GM, French CJ, Deane AM, Shehabi Y, Hajjar LA, Oliveira G, Udy AA, Orford N, Edney SJ, Hunt AL, Judd HL, Bitker L, Cioccari L, Naorungroj T, Yanase F, Bates S, McGain F, Hudson EP, Al-Bassam W, Dwivedi DB, Peppin C, McCracken P, Orosz J, Bailey M, Bellomo R, VITAMINS Trial Investigators (2020). Effect of Vitamin C, Hydrocortisone, and Thiamine vs
Hydrocortisone Alone on Time Alive and Free of Vasopressor Support Among
Patients With Septic Shock: The VITAMINS Randomized Clinical
Trial. JAMA.

[17] Ferreira FL, Bota DP, Bross A, Mélot C, Vincent JL (2001). Serial evaluation of the SOFA score to predict outcome in
critically ill patients. JAMA.

[18] Joffre J, Hellman J, Ince C, Ait-Oufella H (2020). Endothelial responses in sepsis. Am J Respir Crit Care Med.

[19] Wong BJ, Wilkins BW, Holowatz LA, Minson CT (2003). Nitric oxide synthase inhibition does not alter the reactive
hyperemic response in the cutaneous circulation. J Appl Physiol.

[20] Hellmann M, Gaillard-Bigot F, Roustit M, Cracowski JL (2015). Prostanoids are not involved in postocclusive reactive hyperaemia
in human skin. Fundam Clin Pharmacol.

[21] Cracowski JL, Gaillard-Bigot F, Cracowski C, Sors C, Roustit M, Millet C (2013). Involvement of cytochrome epoxygenase metabolites in cutaneous
postocclusive hyperemia in humans. J Appl Physiol.

[22] Cracowski JL, Lorenzo S, Minson CT (2007). Effects of local anaesthesia on subdermal needle insertion pain
and subsequent tests of microvascular function in human. Eur J Pharmacol.

[23] Harrison S, Geppetti P (2001). Substance P. Int J Biochem Cell Biol.

[24] Weidner C, Klede M, Rukwied R, Lischetzki G, Neisius U, Skov PS (2000). Acute effects of substance P and calcitonin gene-related peptide
in human skin--a microdialysis study. J Invest Dermatol.

